# Clinical utility of the RACE score for differentiating stroke from stroke mimics in the emergency department

**DOI:** 10.3389/fneur.2026.1832156

**Published:** 2026-04-29

**Authors:** Hatice Yelda Yildiz, Kaan Gülcan, Süreyya Ece Kozba, İrem Özürün, Zeynep Demirkiran, Yakup Krespi

**Affiliations:** 1 Department of Neurology, Faculty of Medicine, İstinye University, Istanbul, Türkiye; 2 Fatih District Health Directorate, Fatih, Istanbul, Türkiye; 3 Department of Emergency Medicine, Faculty of Medicine, İstinye University, Istanbul, Türkiye; 4 Department of Anesthesiology and Reanimation, Sultan Abdulhamid Han Training and Research Hospital, Istanbul, Türkiye

**Keywords:** emergency department, large vessel occlusion, RACE score, stroke, stroke mimic

## Abstract

**Objective:**

Rapid differentiation of true stroke from stroke mimics remains a major challenge during emergency department stroke evaluations. This study aimed to evaluate the clinical utility of the Rapid Arterial Occlusion Evaluation (RACE) score in distinguishing stroke from stroke mimics and identifying large vessel occlusion (LVO) in patients evaluated through an emergency department stroke activation workflow.

**Materials and methods:**

This retrospective observational study analyzed routinely collected clinical data from consecutive adult patients evaluated through an emergency department stroke activation pathway. The RACE score was calculated at bedside as part of routine stroke workflow, and final diagnoses were established by vascular neurologists based on clinical evaluation and neuroimaging findings. Patients were classified as stroke or stroke mimic cases. The diagnostic performance of the RACE score for identifying LVO was assessed using receiver operating characteristic (ROC) curve analysis, and logistic regression was used to evaluate the association between RACE score and LVO.

**Results:**

A total of 303 patients were included in the final analysis, of whom 133 (43.9%) were diagnosed with stroke and 170 (56.1%) were classified as stroke mimics. Patients with stroke were significantly older than those with stroke mimics (69.13 ± 12.59 vs. 61.67 ± 17.72 years, *p* = 0.001). The mean RACE score was significantly higher in stroke patients than in stroke mimics (3.15 ± 2.62 vs. 1.64 ± 1.91, *p* < 0.001), and RACE scores ≥5 were more frequent in stroke cases (30.8% vs. 8.8%, *p* < 0.001). LVO was identified in 46 patients (15.4%). Patients with LVO had significantly higher RACE scores than those without LVO (4.59 ± 2.63 vs. 1.85 ± 2.03, *p* < 0.001), and 56.5% of LVO cases had RACE scores ≥5. Logistic regression analysis showed that higher RACE scores were significantly associated with the presence of LVO [odds ratio (OR) 1.59, 95% Confidence Interval (CI) 1.38–1.83, *p* < 0.001].

**Conclusion:**

The RACE score may provide clinically useful information for differentiating stroke from stroke mimics during emergency department evaluations. Higher RACE scores were associated with confirmed stroke and the presence of LVO, suggesting that the RACE score may serve as a practical adjunct to bedside neurological assessment within acute stroke workflows.

## 1 Introduction

Stroke is a time-critical neurological emergency in which rapid and accurate diagnosis directly influences treatment decisions and clinical outcomes. In the acute setting, distinguishing true stroke from stroke mimics remains a major diagnostic challenge, particularly during early evaluation in the emergency department. This distinction is critically important, as therapeutic decisions such as intravenous thrombolysis and endovascular treatment must often be made within a narrow time window, where delays or misclassification may lead to missed treatment opportunities or unnecessary interventions. From a global perspective, stroke remains the second leading cause of death and the third leading cause of morbidity among adults worldwide, including in Türkiye. According to the 2022 Global Stroke Report published by the World Stroke Organization, approximately one in four individuals over the age of 25 will experience a stroke during their lifetime. This high lifetime risk places a substantial burden not only on affected individuals but also on healthcare systems and national economies. Indeed, the global economic cost attributable to stroke is estimated to exceed 721 billion US dollars (1).

In emergency department stroke evaluations, rapid differentiation between true stroke and stroke mimics represents a major diagnostic challenge. Stroke mimics are frequently encountered among patients presenting with acute neurological symptoms and may include seizures, metabolic disturbances, systemic illnesses, migraine, functional neurological disorders, and other neurological or non-neurological conditions. Previous studies suggest that stroke mimics may account for up to 30–50% of suspected stroke presentations in emergency settings, potentially leading to diagnostic uncertainty and unnecessary activation of stroke pathways (2). Previous studies have shown that prehospital stroke scales are primarily designed to identify LVO and guide triage decisions in acute stroke pathways (3).

Large vessel occlusion (LVO) represents one of the most severe forms of ischemic stroke and is associated with high mortality and disability if not treated promptly. Recent studies have also emphasized the clinical importance of timely identification of vessel occlusion patterns and their impact on treatment outcomes in acute ischemic stroke (4, 5). Advances in mechanical thrombectomy have substantially improved outcomes in selected patients with LVO (6–9). Consequently, considerable attention has been directed toward rapid identification of patients with severe stroke presentations who may benefit from early specialized stroke care.

Although the National Institutes of Health Stroke Scale (NIHSS) remains the most widely accepted tool for assessing stroke severity, its complexity and the level of training required for reliable application limit its use in some emergency and prehospital settings (10). Consequently, several simplified neurological scoring systems have been developed to facilitate rapid clinical assessment of suspected stroke patients. These include the Los Angeles Motor Scale (LAMS) (11), Field Assessment Stroke Triage for Emergency Destination (FAST-ED) (3), Prehospital Acute Stroke Severity Scale (PASS) (12), Cincinnati Prehospital Stroke Scale (CPSS) (13), Cincinnati Stroke Triage Assessment Tool (C-STAT), and the 3-Item Stroke Scale (3ISS) (14). Importantly, these simplified scales are not intended to provide a comprehensive assessment of stroke severity like the NIHSS, but are purpose-built triage tools designed to identify patients with suspected severe stroke, particularly LVO, and to support rapid decision-making in acute stroke pathways. While these scales provide varying levels of sensitivity and specificity for identifying severe stroke presentations, their diagnostic performance remains heterogeneous.

The Rapid Arterial Occlusion Evaluation (RACE) score, introduced by Pérez de la Ossa and colleagues in 2014, is a structured neurological assessment tool based on key focal neurological deficits including facial paralysis, motor weakness, gaze deviation, and cortical dysfunction such as aphasia or agnosia (15). The score was originally developed to identify severe stroke presentations suggestive of LVO. However, because the RACE score captures core neurological deficits commonly observed in acute stroke, its score distribution may also differ between true stroke and stroke mimic presentations.

Despite the widespread use of RACE in acute stroke assessment, most previous studies have primarily focused on its ability to detect LVO rather than its potential role in differentiating stroke from stroke mimics. Furthermore, detailed analyses evaluating how RACE scores behave across different stroke mimic subgroups remain limited. Therefore, the present study aimed to evaluate the clinical utility of the RACE score in distinguishing stroke from stroke mimics among patients evaluated through an emergency department stroke activation workflow.

## 2 Materials and methods

### 2.1 Study design and setting

This study was designed as a retrospective observational analysis of routinely collected clinical data from a comprehensive stroke center at the İstinye University Faculty of Medicine Training and Research Hospital in Istanbul, Türkiye. The center provides emergency stroke care for a metropolitan population of approximately 5–6 million people and covers six service regions within Istanbul while also accepting inter-hospital transfers from surrounding provinces.

The center manages approximately 1,000–1,500 suspected stroke admissions annually. Patients are referred either directly through the national emergency medical services (EMS) system [112 Emergency medical services (EMS)] or through teleconsultation requests from regional hospitals, subject to approval by the on-call vascular neurologist.

The present study analyzed consecutive patients evaluated through the emergency department stroke activation pathway during the study period.

### 2.2 Use of the RACE score in routine stroke workflow

At the study center, RACE score was incorporated into routine bedside neurological assessment within the acute stroke workflow. A Turkish-language clinical form of the RACE score was used in daily practice and had been reviewed by experienced stroke neurologists prior to its integration into the clinical workflow.

Importantly, the RACE score was not implemented specifically for research purposes but was part of routine clinical evaluation for patients undergoing neurocode activation. As a result, RACE assessments were available for a broad spectrum of patients presenting with suspected stroke, including both confirmed stroke cases and stroke mimics.

The RACE score ranges from 0 to 9, with higher scores indicating more severe neurological deficits.

### 2.3 Training and clinical integration

The RACE score was applied by senior medical students participating in a 10-day clinical internship at the stroke center. This internship forms part of the 6th-year clinical training program of the İstinye University Faculty of Medicine and was implemented in 2023 following approval by the university senate.

At the beginning of the internship, students received structured theoretical training covering acute stroke pathophysiology, focused neurological examination techniques, and vascular neuroanatomy. During their clinical rotation, students were integrated into the neurocode workflow under supervision and performed structured bedside neurological assessments, including RACE scoring. This supervised approach enabled consistent application of the scoring system during routine stroke evaluations.

### 2.4 Acute stroke workflow and patient management

Patient management followed the hospital's standardized NEUROCODE Acute Stroke Diagnosis and Treatment Procedure (Document No: INM-P01), initially introduced in 2017 and updated in 2022.

Patients with suspected stroke—either transported by EMS or identified directly in the emergency department—were evaluated through the neurocode activation system. The workflow was coordinated by a multidisciplinary stroke team led by a vascular neurologist and included a stroke practitioner with at least 6 months of stroke care experience as well as a neurocode nurse responsible for coordinating the stroke pathway.

The protocol follows an inclusive approach in which most neurocode-activated patients undergo urgent neuroimaging to confirm or exclude stroke.

The stroke activation pathway at our center is based on a highly sensitive and inclusive approach, in which patients presenting with any acute neurological symptoms suggestive of stroke—such as sudden weakness, speech disturbance, altered consciousness, or unclear neurological deficits—are eligible for neurocode activation. This strategy prioritizes early detection of potential stroke cases and minimizes the risk of missed diagnoses, even at the expense of including a higher proportion of stroke mimic cases.

### 2.5 Application of RACE

Within the neurocode workflow, patients presenting with suspected stroke underwent standardized neurological assessment. The RACE score was routinely calculated at bedside by a neurology intern or supervised medical student, typically during patient transfer from the emergency department to the radiology unit.

The score was applied without prior diagnostic exclusion, ensuring that RACE values were recorded for nearly all neurocode-activated patients, including those subsequently diagnosed as stroke mimics.

### 2.6 Diagnostic confirmation and case classification

Definitive diagnosis was established through a combination of clinical evaluation by a vascular neurologist and neuroimaging findings, including non-contrast computed tomography (CT), CT angiography, and magnetic resonance angiography when clinically indicated.

Based on imaging findings and clinical evaluation, patients were classified as stroke or stroke mimic. Stroke cases were further categorized as ischemic or hemorrhagic.

Stroke mimic cases were subsequently classified according to etiology (neurological vs. non-neurological) and clinical severity using information extracted from emergency department records. Clinical severity classification was based on the need for hospital admission, intensive care support, or urgent specialist intervention.

For subgroup analyses, stroke mimic cases were categorized into four predefined groups based on etiology and clinical severity: neuro-critical (neurological conditions requiring urgent or intensive management), neuro-non-critical (neurological conditions not requiring critical care), non-neuro-critical (non-neurological conditions associated with critical systemic illness requiring urgent intervention), and non-neuro-non-critical (non-neurological conditions not associated with critical illness).

Neurological mimics included conditions such as seizures, migraine, functional neurological disorders, and other acute neurological presentations not attributable to stroke. Non-neurological mimics comprised systemic or metabolic conditions presenting with stroke-like symptoms.

Critical conditions were defined as cases requiring hospital admission, intensive care support, or urgent specialist intervention, whereas non-critical cases were managed with outpatient follow-up or short-term observation. Classification of stroke mimic subgroups was performed retrospectively based on final clinical diagnoses documented in hospital records.

Recorded variables included age, sex, presenting symptoms, RACE score components, stroke subtype, presence of LVO, and anatomical localization. LVO was defined as occlusion of the intracranial internal carotid artery (ICA), the M1 segment of the middle cerebral artery, or the basilar artery. All data were retrieved from the secure institutional stroke registry database of the İstinye University Stroke Center.

Importantly, the RACE score was not used as a diagnostic criterion in determining the final diagnosis. Diagnostic classification was performed independently by vascular neurologists based on comprehensive clinical evaluation and neuroimaging findings.

### 2.7 Eligibility criteria

Patients younger than 18 years of age, those with a documented history of previous stroke (to avoid potential influence of residual neurological deficits on RACE scoring), patients presenting with trauma-related neurological symptoms, and cases with incomplete clinical or imaging data were excluded from the analysis.

### 2.8 Statistical analyses and tools

Statistical analyses were performed using Wistats v3.0 (WisdomEra Corp., Istanbul, Türkiye), a Python-based statistical analysis platform utilizing established scientific libraries including SciPy v1.2.3, scikit-learn v0.24.0, and statsmodels v0.9.0.

Data distribution was evaluated using skewness and kurtosis statistics and confirmed with the Shapiro–Wilk test. Selection of statistical tests for group comparisons was based on distribution characteristics.

Categorical variables were compared using Chi-square or Fisher's exact tests when appropriate. Comparisons of continuous variables between two groups were performed using either the Independent Samples *t*-test for normally distributed data or the Mann–Whitney *U*-test for non-normally distributed data. For comparisons involving more than two groups, one-way ANOVA or Kruskal–Wallis tests were applied as appropriate.

The primary analysis compared RACE score distributions between stroke and stroke mimic groups. Additional analyses evaluated differences in clinical characteristics and RACE scores according to LVO status.

Univariate logistic regression analysis was performed to evaluate the association between the RACE score and the presence of LVO. The diagnostic performance of the RACE score for identifying LVO was further evaluated using receiver operating characteristic (ROC) curve analysis. The area under the curve (AUC) was calculated to assess discriminative ability. The optimal cutoff value was determined using the Youden index (sensitivity + specificity – 1).

Sensitivity, specificity, positive predictive value (PPV), and negative predictive value (NPV) were calculated for different RACE score thresholds. Two-tailed *p* values < 0.05 were considered statistically significant.

## 3 Results

### 3.1 Study population and baseline characteristics

**Figure 1:**
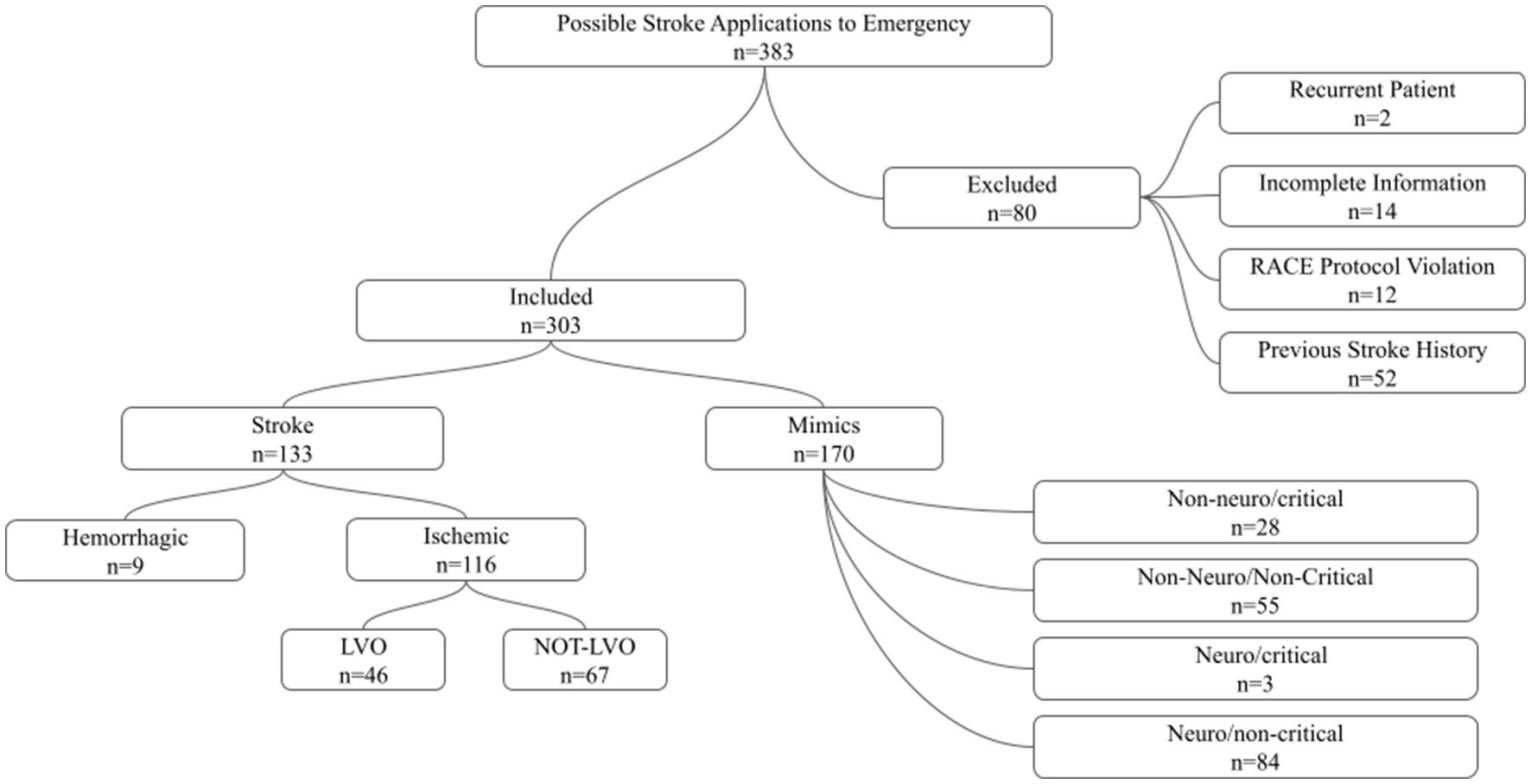
Study flow diagram and distribution of patient groups. Among 383 patients presenting to the emergency department with suspected stroke, 80 were excluded due to recurrent presentation, incomplete information, RACE protocol violation, or previous stroke history. The final cohort included 303 patients, of whom 133 were diagnosed with stroke and 170 were classified as stroke mimics. Stroke cases were further categorized as ischemic or hemorrhagic, and ischemic strokes were stratified according to the presence or absence of large vessel occlusion (LVO). Stroke mimic cases were classified according to etiology and clinical severity.

A total of 303 patients were included in the final analysis after applying the predefined exclusion criteria (Figure 1). Among these patients, 170 (56.1%) were classified as stroke mimics and 133 (43.9%) were diagnosed with stroke. The overall study population had a mean age of 64.95 ± 16.08 years, and 52.1% of patients were male (Table 1).

**Table 1:** Baseline characteristics of the study population.

Variables	Cases Mean ±SD, *N* (%)
***N***	303 (100%)
Sex	
Female	145 (47.9%)
Male	158 (52.1%)
Age	64.95 ± 16.08
Case group	
Stroke mimics	170 (56.1%)
Stroke	133 (43.9%)
Stroke mimics group	
Neuro-critical	3 (1.8%)
Neuro-non-critical	84 (49.4%)
Non-neuro-non-critical	55 (32.4%)
Non-neuro-critical	28 (16.5%)
Stroke type	
Hemorrhagic	9 (7.2%)
Ischemic	116 (92.8%)
Stroke mimics neurological	
No	83 (48.8%)
Yes	87 (51.2%)
Large vessel occlusion	
No	257 (84.6%)
Yes	46 (15.4%)
Oxfordshire community stroke project	
LACS	18 (14.4%)
PACS	51 (40.8%)
POCS	29 (23.2%)
TACS	27 (21.6%)
Facial palsy score	
0	170 (56.1%)
1	99 (32.7%)
2	34 (11.2%)
Arm motor impairment	
0	186 (61.4%)
1	71 (23.4%)
2	46 (15.2%)
Leg motor impairment	
0	178 (58.7%)
1	73 (24.1%)
2	52 (17.2%)
Head and gaze deviation	
0	264 (88.3%)
1	33 (11.0%)
2	2 (0.7%)
Aphasia score	
0	193 (67.7%)
1	58 (20.4%)
2	34 (11.9%)
Agnosia score	
0	191 (91.4%)
1	11 (5.3%)
2	7 (3.3%)
**RACE score**	2.30 ± 2.37
RACE score group	
< 5	247 (81.5%)
≥5	56 (18.5%)

N, number of cases; RACE, rapid arterial occlusion evaluation; LACS, lacunar syndrome; PACS, partial anterior circulation syndrome; POCS, posterior circulation syndrome; TACS, total anterior circulation syndrome; SD, standard deviation.

Within the stroke group, ischemic stroke accounted for 92.8% (*n* = 116) of cases, whereas hemorrhagic stroke constituted 7.2% (*n* = 9). Stroke mimic cases were categorized into four groups: neuro-non-critical (49.4%), non-neuro-non-critical (32.4%), non-neuro-critical (16.5%), and neuro-critical (1.8%).

LVO was identified in 46 patients (15.4%), while 253 patients (84.6%) had no LVO. The mean RACE score in the overall cohort was 2.30 ± 2.37, and 18.5% of patients had a RACE score ≥5.

### 3.2 Comparison between stroke and stroke mimic cases

Patients with stroke were significantly older than those with stroke mimics (69.13 ± 12.59 vs. 61.67 ± 17.72 years, *p* = 0.001). Sex distribution did not differ significantly between the groups (*p* = 0.487; Table 2).

**Table 2:** Comparison of clinical characteristics and RACE scores between stroke and stroke mimic cases.

Variables	Stroke mimics	Stroke	*p*
	Mean ±*SD*, *N* (%)	Mean ±*SD*, *N* (%)	
***N***	170 (56.1%)	133 (43.9%)	
Sex			
Female	82 (48.2%)	63 (47.4%)	0.487
Male	88 (51.8%)	70 (52.6%)	
**Age**	61.67 ± 17.72	69.13 ± 12.59	**0.001**
Facial palsy score			
0	119 (70.0%)	51 (38.3%)	**< 0.001**
1	40 (23.5%)	59 (44.4%)	
2	11 (6.5%)	23 (17.3%)	
Arm motor impairment			
0	123 (72.4%)	63 (47.4%)	**< 0.001**
1	33 (19.4%)	38 (28.6%)	
2	14 (8.2%)	32 (24.1%)	
Leg motor impairment			
0	113 (66.5%)	65 (48.9%)	**0.005**
1	36 (21.2%)	37 (27.8%)	
2	21 (12.4%)	31 (23.3%)	
Head and gaze deviation			
0	160 (96.4%)	104 (78.2%)	**< 0.001**
1	6 (3.6%)	27 (20.3%)	
2	0 (0.0%)	2 (1.5%)	
Aphasia score			
0	118 (74.2%)	75 (59.5%)	**0.020**
1	28 (17.6%)	30 (23.8%)	
2	13 (8.2%)	21 (16.7%)	
Agnosia score			
0	116 (91.3%)	75 (91.5%)	0.962
1	7 (5.5%)	4 (4.9%)	
2	4 (3.1%)	3 (3.7%)	
**RACE score**	1.64 ± 1.91	3.15 ± 2.62	**< 0.001**
RACE score group			
< 5	155 (91.2%)	92 (69.2%)	**< 0.001**
≥5	15 (8.8%)	41 (30.8%)	

N, Number of cases; RACE, rapid arterial occlusion evaluation; SD, standard deviation. Bold values indicate statistically significant results (*p* < 0.05).

Neurological deficits captured by individual RACE components were significantly more frequent among stroke cases. Facial palsy scores differed significantly between the groups (*p* < 0.001). Similarly, brachial paresis (*p* < 0.001), crural paresis (*p* = 0.005), and oculocephalic deviation (*p* < 0.001) were more severe among stroke patients. Aphasia scores showed a modest but statistically significant difference (*p* = 0.040), whereas agnosia scores did not differ significantly (*p* = 0.818).

**Figure 2:**
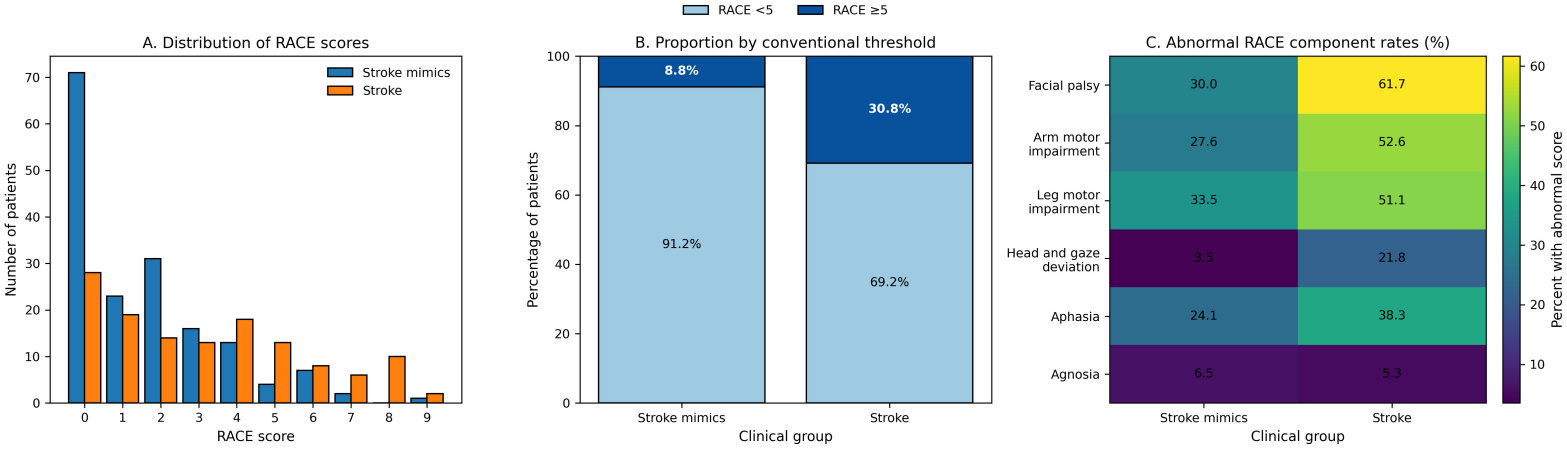
RACE score profile in stroke and stroke mimic cases. **(A)** Distribution of RACE scores among patients diagnosed with stroke and stroke mimics. Higher RACE scores were observed more frequently among stroke cases, whereas lower scores were predominantly seen in stroke mimics. **(B)** Proportion of patients according to the conventional RACE threshold (< 5 and ≥5). A substantially higher proportion of stroke patients had RACE scores ≥5 compared with stroke mimic cases. **(C)** Heatmap showing the percentage of patients with abnormal findings for individual RACE components in stroke and stroke mimic groups. Motor deficits and gaze deviation were markedly more frequent among stroke cases, whereas these findings were less common in stroke mimics.

The overall RACE score was significantly higher in stroke patients than in stroke mimics (3.15 ± 2.62 vs. 1.64 ± 1.91, *p* < 0.001; Figure 2). When categorized according to the conventional threshold, 30.8% of stroke patients had a RACE score ≥5 compared with 8.8% of stroke mimic cases (*p* < 0.001).

### 3.3 Comparison according to large vessel occlusion status

All major neurological deficit components included in the RACE score—facial palsy, brachial paresis, crural paresis, oculocephalic deviation, and aphasia—were significantly more severe among patients with LVO (all *p* < 0.001), whereas agnosia scores did not differ significantly (*p* = 0.458; Table 3).

**Table 3:** Comparison of clinical characteristics and RACE scores between patients with and without large vessel occlusion.

Variables	No^*^	Yes	*p*
Mean ±*SD*, *N* (%)	Mean ±*SD*, *N* (%)
***N***	253 (84.6)	46 (15.4)	
Facial palsy score			
0	158 (62.5%)	11 (23.9%)	**< 0.001**
1	72 (28.5%)	26 (56.5%)	
2	23 (9.1%)	9 (19.6%)	
Arm motor impairment			
0	174 (68.8%)	11 (23.9%)	**< 0.001**
1	56 (22.1%)	14 (30.4%)	
2	23 (9.1%)	21 (45.7%)	
Leg motor impairment			
0	163 (64.4%)	13 (28.3%)	**< 0.001**
1	60 (23.7%)	13 (28.3%)	
2	30 (11.9%)	20 (43.5%)	
Head and gaze deviation			
0	229 (92.0%)	32 (69.6%)	**< 0.001**
1	20 (8.0%)	12 (26.1%)	
2	0 (0.0%)	2 (4.3%)	
Aphasia score			
0	171 (71.5%)	19 (45.2%)	**< 0.001**
1	47 (19.7%)	11 (26.2%)	
2	21 (8.8%)	12 (28.6%)	
Agnosia score			
0	169 (92.3%)	19 (82.6%)	**0.025**
1	10 (5.5%)	1 (4.3%)	
2	4 (2.2%)	3 (13.0%)	
**RACE score**	1.85 ± 2.03	4.59 ± 2.63	**< 0.001**
RACE score group			
< 5	225 (88.9%)	20 (43.5%)	**< 0.001**
≥5	28 (11.1%)	26 (56.5%)	

N, number of cases; RACE, rapid arterial occlusion evaluation; SD, standard deviation.

^*^Patients without large vessel occlusion (LVO) include both non-LVO stroke cases and stroke mimic presentations. Bold values indicate statistically significant results (*p* < 0.05).

**Figure 3:**
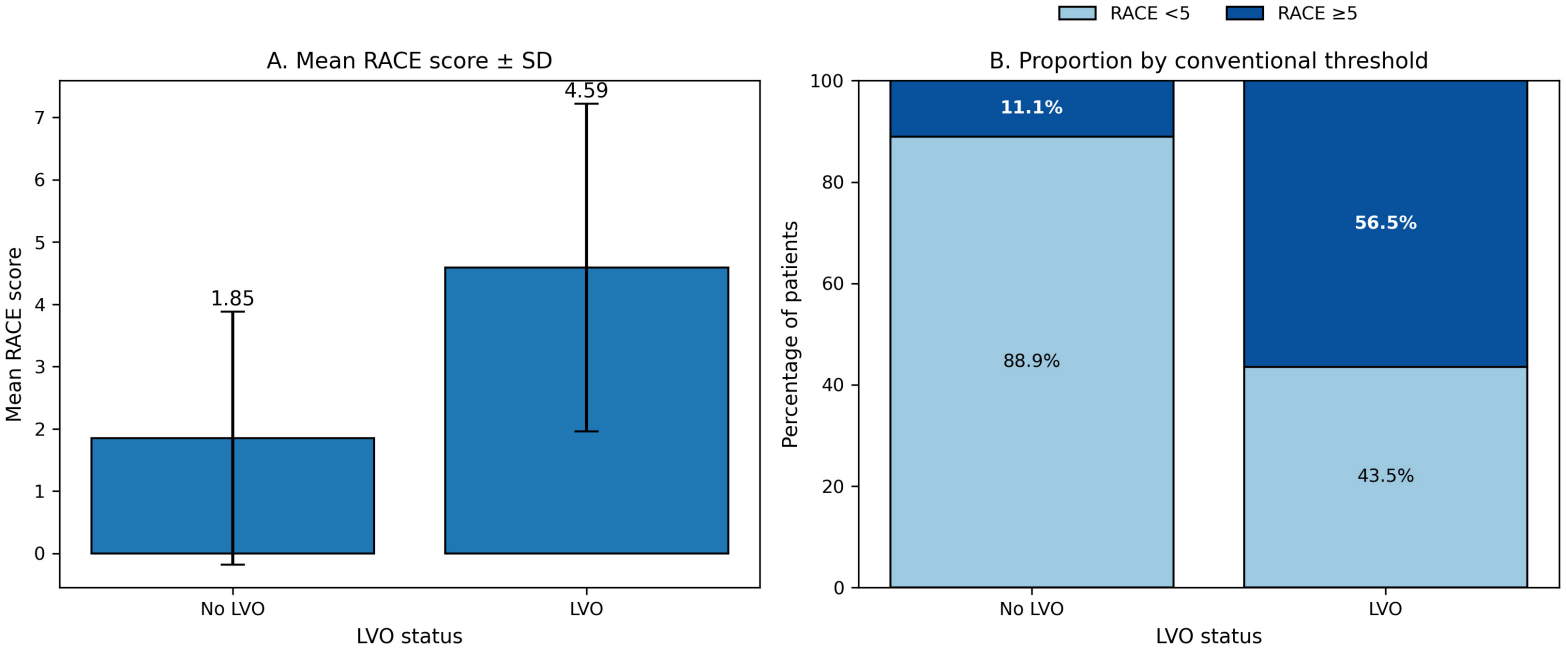
RACE score characteristics according to large vessel occlusion (LVO) status. **(A)** Mean RACE scores in patients with and without large vessel occlusion. Patients with LVO demonstrated markedly higher RACE scores compared with those without LVO. **(B)** Proportion of patients with RACE scores < 5 and ≥5 according to LVO status. More than half of the patients with LVO had RACE scores ≥5, whereas the majority of patients without LVO had scores below this threshold.

The mean RACE score was significantly higher in patients with LVO than in those without LVO (4.59 ± 2.63 vs. 1.85 ± 2.03, *p* < 0.001; Figure 3). When categorized using the conventional cutoff value, 56.5% of LVO patients had RACE scores ≥5 compared with 11.1% of patients without LVO (*p* < 0.001).

### 3.4 Predictors of large vessel occlusion

Univariate logistic regression analysis demonstrated a significant association between the RACE score and the presence of LVO. Each one-point increase in the RACE score was associated with a higher likelihood of LVO [odds ratio (OR) 1.59, 95% confidence interval (CI) 1.38–1.83, *p* < 0.001].

**Figure 4:**
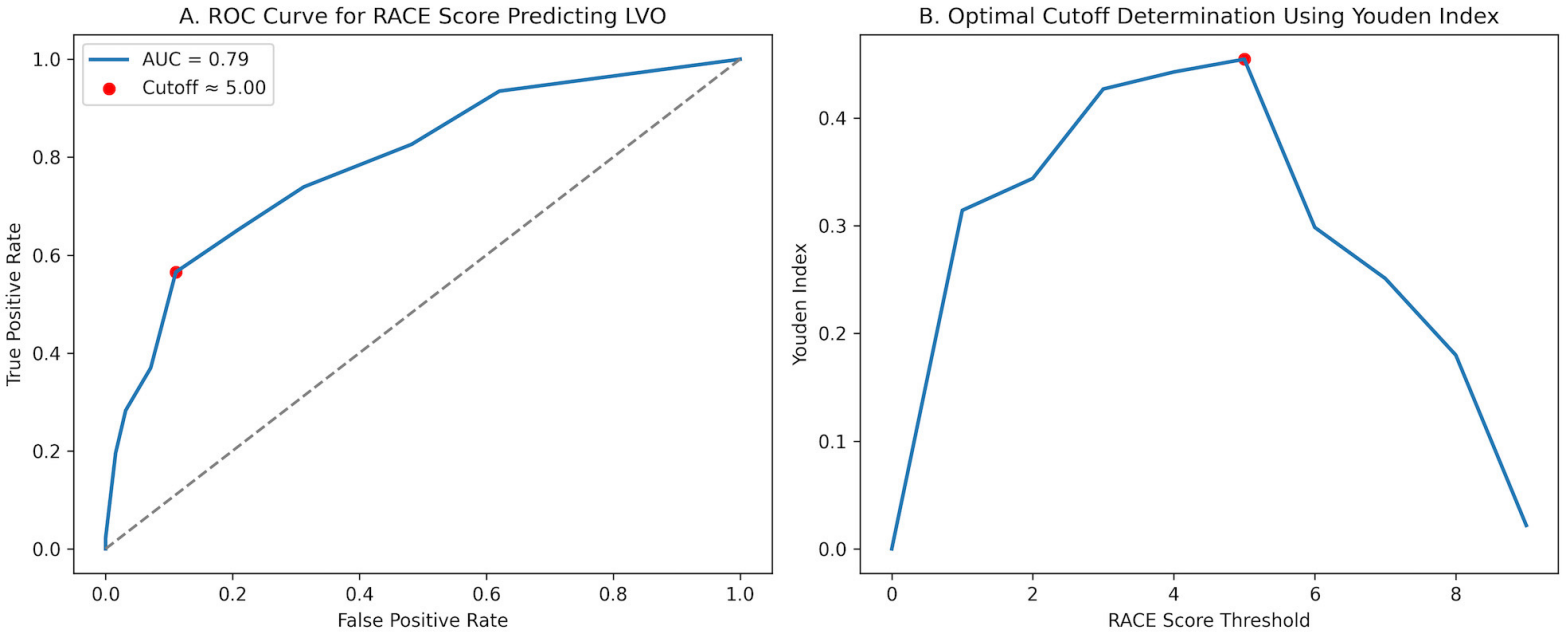
Diagnostic performance of the RACE score for identifying large vessel occlusion. **(A)** Receiver operating characteristic (ROC) curve demonstrating the ability of the rapid arterial occlusion evaluation (RACE) score to identify large vessel occlusion (LVO). The area under the curve (AUC) represents the overall discriminative performance of the score. The highlighted point indicates the optimal cutoff value determined by the Youden index. **(B)** Determination of the optimal RACE score threshold using the Youden index (sensitivity + specificity – 1). The maximum Youden index identifies the cutoff that provides the best balance between sensitivity and specificity for detecting LVO.

The diagnostic performance of the RACE score for identifying LVO was further evaluated using receiver operating characteristic (ROC) curve analysis (Figure 4). The area under the ROC curve demonstrated good discriminative ability of the RACE score for detecting LVO. The optimal cutoff value determined using the Youden index corresponded to the conventional threshold of RACE ≥5.

## 4 Discussion

This study evaluated the clinical utility of the RACE score in differentiating stroke from stroke mimics within an emergency department stroke activation workflow. Overall, RACE scores were significantly higher in patients with true stroke compared with stroke mimics, supporting the potential diagnostic value of structured neurological deficit scoring during early stroke evaluation. In the present cohort, patients diagnosed with stroke had significantly higher RACE scores than mimic cases, and RACE scores ≥5 were substantially more frequent among stroke patients. These findings suggest that the distribution of RACE scores may provide useful clinical information in distinguishing true stroke presentations from stroke mimics in the acute setting.

In addition to stroke–mimic differentiation, the RACE score also demonstrated strong performance in identifying LVO. Patients with LVO had markedly higher RACE scores compared with patients without LVO, and more than half of LVO cases had RACE scores ≥5. This observation is consistent with the original design of the RACE score as a clinical tool for identifying severe stroke presentations suggestive of LVO. The discriminative capacity observed in this study aligns with previous validation studies that reported moderate-to-good diagnostic performance of RACE for LVO detection (16, 17). In particular, the relatively high specificity observed at the conventional threshold of ≥5 supports the usefulness of RACE for identifying patients who may benefit from rapid transfer to comprehensive stroke centers and advanced reperfusion therapies (18).

An important finding of the present study is the strong association between the RACE score and the presence of LVO. In logistic regression analysis, higher RACE scores were significantly associated with increased likelihood of LVO. This observation supports the clinical rationale underlying the design of the RACE score, which incorporates key neurological deficits such as gaze deviation, motor weakness, and cortical dysfunction that are commonly observed in severe anterior circulation strokes. Although comprehensive neurological severity scales such as the NIHSS are widely used to quantify overall stroke severity, simplified scales such as the RACE score focus on selected cortical and motor deficits that are more directly associated with LVO. This targeted approach may explain their practical utility in supporting rapid triage decisions in acute stroke pathways.

The practical performance of the RACE score should also be interpreted in the context of its implementation. Structured clinical assessment tools are increasingly incorporated into routine stroke workflows to standardize bedside evaluation and improve early clinical decision-making in acute stroke care (19). In this study, RACE assessments were performed by medical students who had received structured training in neurological examination. Early acquisition of standardized neurological assessment skills may facilitate the adoption of structured clinical scoring systems and help overcome the perceived complexity of neurological examination in acute care settings.

Another important aspect of this study is the detailed evaluation of stroke mimic presentations. Stroke mimics represented more than half of the final study cohort, reflecting the broad inclusion strategy used in the emergency stroke pathway. When mimic cases were stratified by etiology and clinical severity, RACE scores generally remained low across most mimic subgroups, particularly in non-critical non-neurological conditions. These findings support the notion that structured neurological deficit scoring systems may contribute not only to identifying severe stroke but also to the early recognition of non-stroke presentations.

This observation is broadly consistent with previous reports showing that stroke mimics constitute a substantial proportion of suspected stroke presentations in acute care pathways (2). At the same time, prior studies of prehospital LVO scales have mainly focused on triage performance rather than differentiation of stroke from stroke mimics, and have reported variable diagnostic accuracy (3, 16). In this context, our finding that RACE scores remained generally low across most mimic subgroups, particularly in non-critical non-neurological conditions, supports the potential practical value of the scale while also highlighting that it should be interpreted as an adjunct rather than a stand-alone diagnostic tool.

In modern comprehensive stroke centers with rapid access to neuroimaging, definitive diagnosis and treatment decisions are primarily guided by imaging findings. Therefore, the RACE score should not be considered a substitute for imaging-based evaluation. Instead, its potential clinical value lies in supporting early bedside assessment, facilitating initial risk stratification, and aiding clinical prioritization before imaging is performed, particularly in busy emergency department settings. However, this study did not evaluate the impact of RACE on workflow metrics such as door-to-imaging or door-to-needle times, and further studies are needed to determine its effect on clinical decision-making and patient outcomes in this context.

Several limitations should be acknowledged. First, this was a single-center study conducted at a comprehensive stroke center, which may limit the generalizability of the findings to healthcare systems with different stroke triage structures or emergency care pathways. Second, RACE assessments were performed by medical students rather than paramedics or emergency medical technicians. Although this design allowed evaluation of the score among non-specialist users, it may not fully reflect real-world prehospital practice where stroke scales are typically applied by emergency medical personnel. Third, other commonly used neurological severity scales, such as the NIHSS and the Glasgow Coma Scale, were not systematically documented for stroke mimic presentations in the emergency workflow and were therefore not included in the comparative analyses. Consequently, direct comparison between the RACE score and other established neurological assessment tools was not possible in the present study. Future studies in which both stroke and stroke mimic patients undergo standardized neurological assessment with multiple scoring systems may provide more comprehensive comparative evidence regarding the diagnostic utility of these tools. Fourth, the number of patients with confirmed LVO was relatively limited, which may influence the precision of the estimated associations between RACE scores and LVO. An important consideration of this study is the relatively high proportion of stroke mimics, which reflects the inclusive nature of the stroke activation pathway at our center. This approach, while increasing sensitivity for detecting true stroke cases, may introduce selection bias and limit the generalizability of the findings to centers with more restrictive triage protocols. Therefore, the diagnostic performance of the RACE score observed in this study should be interpreted in the context of this specific clinical setting. An important clinical consideration is the presence of stroke cases with low RACE scores, representing potential false-negative results. Although the RACE score demonstrated good performance in identifying severe stroke and LVO, it may be less sensitive for detecting minor strokes or posterior circulation events. This limitation is consistent with previous studies of simplified stroke scales and highlights the importance of not relying solely on RACE for diagnostic decision-making. Therefore, low RACE scores should be interpreted with caution, and comprehensive clinical assessment and imaging remain essential to avoid missed diagnoses in acute stroke settings. Finally, outcome measures were limited to diagnostic performance and did not include long-term functional outcomes or system-level workflow metrics.

Nevertheless, an important strength of this study is that RACE assessments were performed as part of routine clinical practice within an operational stroke activation workflow. This allowed evaluation of the score under real-world conditions rather than within a strictly controlled research environment.

Future multicenter studies with larger cohorts may further clarify the role of the RACE score not only in identifying LVO but also in supporting the early differentiation of stroke from stroke mimics in emergency stroke pathways.

## 5 Conclusions

The present study suggests that the RACE score may provide clinically useful information in differentiating stroke from stroke mimics during emergency department stroke evaluations. RACE scores were significantly higher in patients with confirmed stroke compared with stroke mimic cases, indicating that the distribution of neurological deficit scores may support early diagnostic stratification in patients presenting with suspected stroke.

In addition, the RACE score demonstrated strong association with LVO. Higher RACE scores were observed among patients with LVO and were significantly associated with the presence of LVO. These findings support the potential utility of the RACE score not only for identifying severe stroke presentations but also for assisting early triage decisions in acute stroke pathways.

Overall, the RACE score may serve as a practical adjunct to bedside neurological assessment within emergency stroke workflows, helping clinicians rapidly identify patients with a higher likelihood of true stroke and LVO.

## Data Availability

The dataset generated and analyzed in this study is available through the Istinye University Dataset Sharing Platform. Anonymized clinical data can be accessed at the following link: https://dataset.istinye.edu.tr/dataset?did=77. All data were fully anonymized in accordance with ethical regulations. Access is provided for research purposes through a controlled-access system under the platform's standard licensing and data-sharing policies.
